# P-198. Simulation: Advancing Healthcare Worker Education for C. diff

**DOI:** 10.1093/ofid/ofae631.402

**Published:** 2025-01-29

**Authors:** Delvina Ford, Jose Cadena, Lauren N Garza, Dora Marrufo, Debra Bartoshevich, Ana Mella-Davila, Erica Beck

**Affiliations:** South Texas Veterans Health Care System, San Antonio, Texas; South Texas Veterans Health Care System, UT Health San Antonio, San Antonio, Texas; South Texas Veteran Health Care System, San Antonio, Texas; South Texas Veterans Healthcare System, San Antonio, Texas; South Texas Veterans Healthcare System, San Antonio, Texas; South Texas Veterans Healthcare System, San Antonio, Texas; Bitscopic, Inc., San Antonio, Texas

## Abstract

**Background:**

Use of scenario-based simulation for healthcare personal (HCP) in the prevention of healthcare associated infections remains an area that is proving to have positive educational effects. The Society for Healthcare Epidemiology of America (SHEA) 2022 Recommendations ongoing assessment and reinforced education of CDI knowledge for HCPs.

**Figure:**
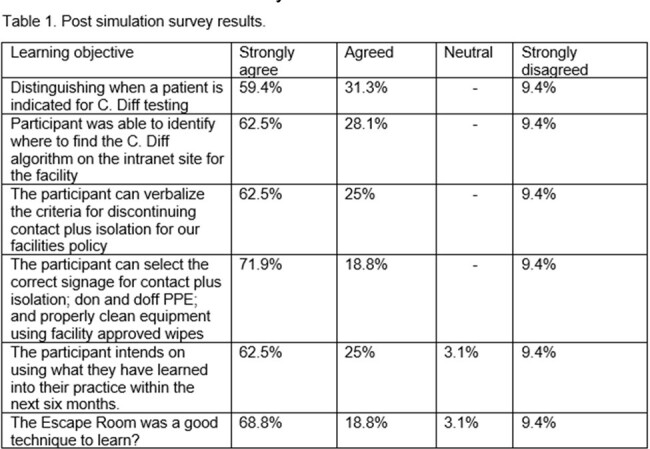

**Methods:**

This simulation was developed as a learning intervention to increase the functional skills of bedside HCPs. An interdisciplinary team created a thirty-minute escape room theme for personal protective equipment, diagnostic stewardship processes.

Focused objectives included C. difficile laboratory testing indications, functional ability to locate the facility algorithm for collection and testing, direct hands-on use of personal protective equipment, hospital disinfectant use, appropriate hand hygiene, and the correct use of isolation signage for the facility was demonstrated.

HCP participants were sent a post participation evaluation utilizing a Likert Scale.

Staff Attendance During First Week of Simulation for C.diff Escape Room

**Figure:**
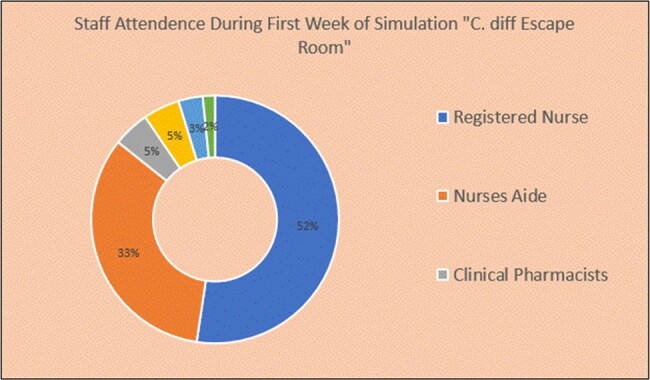

Staff Attendance During First Week of Simulation for C.diff Escape Room

**Results:**

A total of sixty-three HCP participated in the first week. Thirty-three registered nurses (52%), twenty-one nurses’ aides (33%), three clinical pharmacists (5%), three registered cardiovascular invasive specialists (5%), two health technicians (3%), and one licensed vocational nurse (2%).

Post evaluation of the ”C. diff Escape Room” from thirty-two responses of the sixty-three HCP that participated. (see table 1).

Most participants found the scenario-based learning useful, and a significant proportion would use the information and integrate it into their practice within the next six months (87.5% agreed/strongly agreed), facilitators were found to be knowledgeable (68% strongly agreed, 21.9% agreed) and made them feel comfortable (68.8% strongly agreed, 18.8% agreed).

**Conclusion:**

Scenario-based simulation was an acceptable method to educate a diverse group of HCP on the diagnosis and isolation management of patients with the evaluation or diagnosis of CDI.

The utilization of the simulation in a fun environment provided for an educational experience with a focus on key objectives for learning. The event was well received, and overall respondents had positive feedback.

**Disclosures:**

**All Authors**: No reported disclosures

